# Anwendung von Zwang in der Intensivmedizin

**DOI:** 10.1007/s00063-021-00800-9

**Published:** 2021-03-03

**Authors:** S. Jöbges, N. Biller-Andorno

**Affiliations:** Institut für Biomedizinische Ethik und Medizingeschichte (IBME), Universität Zürich, Winterthurerstrasse 30, 8006 Zürich, Schweiz

**Keywords:** Persönliche Autonomie, Freiheit, Entscheidungsfindung, Klinische Ethik, Rechtliche Aspekte, Personal autonomy, Freedom, Decision making, Clinical ethics, Legal aspects

## Abstract

Zwangsbehandlungen in der Medizin umfassen Maßnahmen, die gegen eine aktuelle oder frühere Willensäußerung der betroffenen Person durchgeführt werden. Hierunter fällt auch die Überwindung manifestierter Widerstände z. B. bei nicht einwilligungsfähigen Patienten. Zwang gibt es nicht nur in der Psychiatrie, sondern kann auch auf der Intensivstation ausgeübt werden. Im Spannungsfeld zwischen intensivmedizinischer Behandlung, Fürsorge und Patientenwille besteht ein hohes Risiko für Zwangsbehandlungen sowie freiheitseinschränkende Maßnahmen. Häufig ist dem Team dieses moralische Spannungsfeld nur zum Teil bewusst. Vom Patienten wird Zwang als Kontrollverlust beschrieben und kann als traumatisierend, entwürdigend und stressauslösend wahrgenommen werden. Die Herausforderung für das Team einer hochspezialisierten Intensivstation besteht darin, den Patienten in seiner Individualität zu sehen und so weit wie möglich einzubinden. Um Zwang auf Intensivstation zu vermeiden und dem individuellen Patienten gerecht zu werden, muss die Problematik zuallererst wahrgenommen werden. Hilfreich zur Vermeidung von Zwang auf einer Intensivstation können Ausbildungskonzepte, eine ethische Reflexion im Team (Teamkultur), Supervision und psychologische Begleitung für Patienten und das Team sowie klinikinternen Standards sein. Diese Arbeit beschreibt Ursachen, verschiedene Formen und Häufigkeiten von Zwangsbehandlungen auf der Intensivstation sowie juristische Vorgaben. Es wird eine Annäherung versucht, welche intensivmedizinischen Maßnahmen mit der Ausübung von Zwang einhergehen können und wie Zwang von Patienten und dem Team wahrgenommen wird.

## Abstract (en)

Coercive treatment in medicine includes measures taken against a current or previous expression of the will of the person concerned. It can include overcoming manifested resistance, especially in patients who no longer have the capacity to consent. Even though coercive measurements are common in psychiatry, they are also used in intensive care units (ICU). Use of coercive measurements in the ICU has always been a conflict between providing best medical care and restriction of free will/patient will. Medical staff is generally only partially aware of the moral conflict of these measures. However, patients have described coercion as an active loss of free will which they experience to be dehumanizing, stressful and traumatizing. The challenge in the ICU is to focus on the individual needs of the patients and involve them as much as possible while providing high-quality, highly specialized medical care. In order to avoid coersion in the ICU and to do justice to the individual patient, the focus must shift to building awareness. Models that have been shown to improve awareness such as the ethical reflection within the team, supervision and psychological support for patients and internal hospital standards have also been shown to reduce coercive measurements taken. The aim of this paper is to describe causes, different methods and frequencies of coercive measures used in the ICU. Legal aspects are also taken into account. This paper attempts to identify which procedures undertaken in the ICU can be associated with coercive measurements and how coercion is experienced by patients and the team.

## Hintergrund

Oft wird bei dem Thema Ausübung von Zwang in der Medizin zunächst an die Psychiatrie gedacht. Dort setzt man sich seit längerem u. a. in Form von Richtlinien und empirischen Studien mit der Thematik auseinander [1, 2]. Eine Gefahr besteht darin, dass Zwang im Bereich der Intensivmedizin häufig nicht als solcher wahrgenommen wird und damit einer kritischen Reflexion unzugänglich bleibt. Zugleich wird auch in der Intensivmedizin der Respekt vor der Patientenautonomie durchaus hoch gewichtet und versucht, die Vorstellungen nichturteilsfähiger Patienten soweit wie möglich in die Behandlung einzubeziehen.

## Bedeutung des Begriffs Zwang

Der Begriff „Zwang“ (mittelhochdeutsch wanc, dwanc, twanc) umfasst verschiedene Bedeutungen: Zwang als Beschränkung oder Beeinflussung des freien Willens wird unterschieden vom inneren Zwang, vom juristischen Zwang sowie von Zwangsstörungen im Sinne einer psychischen Erkrankung.

Die hier beschriebene Problematik betrifft Zwang als Überwindung des freien Willens bzw. die Überwindung des „natürlichen Willens“ einer Person im Rahmen einer intensivmedizinischen Behandlung. Unter „natürlichem Willen“ werden Willensäußerungen von nichteinwilligungsfähigen Menschen verstanden [1]. Diese Äußerungen in Therapiekonzepte einzubinden, erfordert ein hohes Maß an Fürsorge.

## Juristische Vorgaben in Deutschland

Die Freiheit von äußerlichem Zwang als einem Menschenrecht findet ihre Entsprechung im Grundgesetz Art. 2 Abs. 2, Satz 2, in dem konstatiert wird: „(2) Jeder hat das Recht auf Leben und körperliche Unversehrtheit. Die Freiheit der Person ist unverletzlich. In diese Rechte darf nur auf Grund eines Gesetzes eingegriffen werden.“ [3]. Weiterhin, legt das Grundgesetz Art. 104 fest, dass Menschen „weder seelisch noch körperlich misshandelt werden“ dürfen sowie dass eine richterliche Anordnung über „die Zulässigkeit und Fortdauer einer Freiheitsentziehung“ notwendig ist [4]. Speziell für die Anwendung von körperlicher Fixierung eines Patienten hat das Bundesverfassungsgericht im Jahr 2018 entschieden: „Die Fixierung eines Patienten stellt einen Eingriff in dessen Grundrecht auf Freiheit der Person (Art. 2 Abs. 2 Satz 2 in Verbindung mit Art. 104 GG) dar.“ [5]. Für eine allgemeine stationäre Versorgung legt § 1906a, Bürgerliches Gesetzbuch (BGB), Kriterien für eine Einwilligung in freiheitsbeschränkenden Maßnahmen durch einen Betreuer fest [6]. Hierzu gehören

- das „Patientenwohl“;
- eine „fehlende Einsichtsfähigkeit des Patienten“;
- der nach „§ 1901a zu beachtenden Wille des Betreuten“;
- ein „Versuch einer ernsthaften Überzeugung in die Notwendigkeit der ärztlichen Maßnahmen“;
- der Umstand, dass keine Alternativen bzw. „keine weniger belastende Maßnahme zur Verfügung“ stehen und
- dass der „zu erwartende Nutzen der ärztlichen freiheitsbeschränkenden Maßnahme die zu erwartenden Beeinträchtigungen deutlich überwiegt“. „Die Einwilligung in die ärztliche freiheitsbeschränkende Maßnahme bedarf der Genehmigung des Betreuungsgerichts.“ [6].

## Herausforderung Intensivstation

Intensivmedizinische Therapien mit Einsatz von invasiven und intensiven Behandlungsverfahren kommen bei lebensbedrohlichen Erkrankungen mit Organversagen oder -dysfunktion zum Einsatz. Häufig entwickeln Patienten bedingt durch die schwere Erkrankung, die intensivmedizinischen Maßnahmen und medikamentösen Therapien ein Delir, auch in Kombination mit Aggressivität, das einen erheblichen Einfluss auf den akuten und langfristigen Krankheitsverlauf haben kann [7, 8]. Patienten beschreiben das Durchführen schmerzhafter Prozeduren, das Umgeben sein von Apparaten und Lärm und Schlafmangel in einer Vermischung von Realität und Fiktion [9, 10]. In diesem Kontext einer eingeschränkten Fähigkeit zur autonomen Entscheidung werden Maßnahmen als Zwang gewertet, wenn sie den natürlichen Willen eines Menschen überwinden [11].

Um dem Patienten in der für ihn krisenhaften Situation der Erkrankung und der ggf. resultierenden Urteils- und Einwilligungsunfähigkeit gerecht zu werden, sind Stellvertreterlösungen etabliert [6].

### Ursachen für Zwanganwendung

„Zwang anzuwenden bedeutet, eine Maßnahme durchzuführen, obwohl die davon betroffene Person durch Willensäußerung oder Widerstand kundtut oder früher kundgetan hat, dass sie damit nicht einverstanden ist“ [11].

Den natürlichen Willen eines Menschen überwindende Maßnahmen werden als Zwang gewertet

Im Rahmen einer intensivmedizinischen Behandlung gibt es Situationen, in denen Zwang z. B. durch Medikamentengabe oder Fixierung notwendig erscheint. Freiheitseinschränkende Maßnahmen werden oft mit der Intention eingesetzt, den deliranten, agitierten Patienten zu schützen [7]. Solche freiheitseinschränkenden Maßnahmen erreichen ihr Ziel, den Patienten zu schützen, häufig nicht. Im Gegenteil, in Studien hat sich gezeigt, dass fixierende Maßnahmen zu vermehrten Extubationen durch Patienten sowie zu einem erhöhten Bedarf an sedierenden Medikamenten führen [12, 13]. Ebenso sind Todesfälle im Rahmen nicht korrekt angewandter Fixierungen beschrieben [14].

Beschränkung von Freiheitsrechten auf Intensivstation kann in vielfältiger Form entstehen. In Tab. 1 sind zwangerzeugende Maßnahmen zusammengefasst.

#### Merke.

Körperliche freiheitsbeschränkende Maßnahmen und Prozeduren (= „physical restraints“), die die freie Bewegung des Körpers durch Fixierungen oder angrenzend einschränkende Maßnahmen einschränken [15], können neben einer Fixierung durch Gurte auch Bettgitter, umgebendes Monitoring oder strukturelle Abläufe sowie abgeschlossene Räumlichkeiten, denen der Patient sich nicht entziehen kann, umfassen.

**Table 1:** 

Zwang als Überwindung des Willens	Maßnahmen	Mögliche Manifestation auf der Intensivstation
Direkte oder unmittelbare Einflussnahme	„Physical restraint“ und „Chemical restraint“	Jede Form von Fixierung und bewegungseinschränkenden Maßnahmen Therapien (Physiotherapie, Mobilisation) Medikamentöse Ruhigstellung
Mittelbarer Zwang durch Umgebung mit Einschränkung der Bewegung	„Environmental restraint“	Einschränkung der Bewegungsfreiheit durch Monitoring/Geräte Umgebung (Lärm, Licht, Präsenz und Interaktion)
Faktoren, die zu einem Gefühl des Ausgeliefertseins führen können (erlebte Zwangssituation)	„Psychological restraint“	Abhängigkeit, Schmerzen, Angst Endpersonalisierter Umgang Mangelnde Kommunikation Indirekte – direkte Drohung

Die in 34 europäischen Ländern durchgeführte PRICE-Studie zeigte eine Anwendung von medikamentösen oder mechanischen Fixierungen auf Intensivstationen bei im Mittel 33 % der Patienten. Auffallend sind hier die großen innereuropäischen Unterschiede. So wurde z. B. in Großbritannien und Portugal kein Patient fixiert. Demgegenüber waren in Italien alle beatmeten Patienten fixiert [16]. Fixierungen von 76 % der beatmeten Patienten sind in einer Studie aus Kanada beschrieben [13]. Eine allgemeine Übersicht über fixierende Maßnahmen in Deutschland ergab bei 11,8 % der Patienten (inklusive Intensivstation) freiheitseinschränkende fixierende Maßnahmen [17].

Ins Augenmerk genommen werden sollten auch Maßnahmen, die unter „physical psychological restraint“ – sich einer Situation nicht entziehen können und dem Team ausgeliefert sein – gefasst werden [18]. Zwang ist nicht immer gleichzusetzen mit Gewalt. Auch mittelbare Gewalt, wie verschlossene Türen, Einschränkung der Bewegungsfreiheit und Monitoring, können vom Patienten als Zwang wahrgenommen werden. Befragt man Patienten über ihre Erfahrungen auf der Intensivstation, geben diese auch Einschränkungen der individuelle Freiheit durch Umgebung und Monitoring an [19]. Diese Einschränkung wird auch als „environmental restraint“ beschrieben [18].

#### Merke.

Medikamentöse freiheitsbeschränkende Maßnahmen (= „chemical restraints“) sind Medikamente, die mit dem Ziel eingesetzt werden, den Patienten ruhigzustellen, oder ihn daran hindern, sich fortzubewegen. Somit sind diese Maßnahmen genehmigungspflichtige freiheitsentziehende Maßnahmen [1, 2].

Für eine Einordnung einer Medikamentengabe als freiheitsentziehende Maßnahme ist die Indikation, also der Zweck der Medikamentengabe, entscheidend. So ist der Einsatz von Medikamenten im Rahmen eines Heilzwecks, wie z. B. im Rahmen einer Behandlung eines Delirs auf der Intensivstation, anders zu bewerten, als die medikamentöse Ruhigstellung eines „nervenden“ Patienten. Auch die S3-Leitlinie Analgesie, Sedierung und Delirmanagement in der Intensivmedizin empfiehlt, unnötige Sedierungen zu vermeiden, da sich der Behandlungserfolg hierdurch verschlechtert [8].

#### Merke.

Weniger offensichtlich als körperliche oder medikamentöse Freiheitseinschränkungen sind psychologische freiheitseinschränkende Maßnahmen (= „psychological restraints“). Hierzu zählen Maßnahmen mit psychischer Einflussnahme, wie Drohungen, Fehl- oder Falschinformationen und Manipulation [1, 2, 11], die eine Vorenthaltung von Privilegien und Aktivitäten beinhalten [18].

Die Einschränkung der individuellen Freiheit der eigenen Lebensgestaltung als einer der bedeutendsten Werte der westlichen Zivilisation im Rahmen einer intensivmedizinischen Behandlung wird von vielen Patienten als unangenehm beschrieben [20, 21]. Das Gefühl der Hilflosigkeit, der Verletzlichkeit und des Ausgeliefertseins bestimmt die eigene Wahrnehmung und wird als Kontrollverlust thematisiert [22]. So berichten Patienten, dass die Einschränkung und Unfähigkeit, grundlegende Aktivitäten selbst auszuführen, das Gefühl auslöste „no longer a civilized being“ zu sein [23]. Das Erleiden von Schmerzen mit dem Gefühl der Hilflosigkeit und Verletzung der körperlichen Integrität kann in einer unzureichenden Wahrnehmung und Kommunikation durch das Team begründet sein [19, 24]. Ebenso kann das Gefühl, nicht ernst genommen zu werden, eine aggressive Kommunikation mit indirekten oder direkten Androhungen oder das Nichtdurchführen von Maßnahmen Patienten in Angst versetzen [21, 25].

## Stresssituation für den Patienten

In Verbindung mit freiheitseinschränkenden Maßnahmen werden in der Psychiatrie Trauma und Retraumatisierung, Stress, Angst, das Gefühl, ignoriert zu werden, Kontrollverlust und Dehumanisierung beschrieben [26]. Ähnliche Wahrnehmungen werden von Patienten nach intensivmedizinischem Aufenthalt geschildert. So können freiheitseinschränkende Maßnahmen ein Baustein im Potpourri von Ursachen und Wirkungen der Kurz- und Langzeitfolgen einer intensivmedizinischen Behandlung und eines „post-intensive care syndrome“ (PICS) sein [8]. Patienten in der Ausnahmesituation „Intensivstation“ sind von guter Kommunikation abhängig. Wenn Patienten sich mangelhaft oder falsch informiert fühlen, kann der Eindruck entstehen, als Gegenstand und nicht als Mensch behandelt zu werden [27].

## Rechtfertigende Gründe für Zwang auf der Intensivstation

Ziel einer intensivmedizinischen Behandlung ist es, den Patienten durch eine kritische Phase schwerer krankheits- oder unfallbedingter körperlicher Beeinträchtigungen zu bringen. Auf Seite des Teams liegen das Wissen und vielfältige menschliche und technische Möglichkeiten. Demgegenüber steht ein kritisch kranker Patient, der oft nicht vollständig oder gar nicht einwilligungsfähig ist. Das intensivmedizinische Team übernimmt das „Kommando“ nicht nur für Vitalparameter, sondern häufig auch für den Entscheidungsprozess des Patienten [28]. Hier besteht die Gefahr von paternalistischen Entscheidungen, wenngleich diese vom Team durch Wissen und Fürsorge begründet werden.

Nur in Ausnahmesituationen, wie Notfällen oder Gefährdungssituationen, ist es erlaubt, Behandlungen ohne Einwilligung des Patienten aufgrund des mutmaßlichen Willens durchzuführen [2, 11].

Behandlungen ohne Einwilligung des Patienten sind nur in Ausnahmesituationen erlaubt

Inwieweit freiheitseinschränkende Maßnahmen im Sinne von Zwang, also gegen den Willen des Patienten, zum Erreichen des Therapiezieles notwendig sind, bedarf immer einer individuellen ethischen Reflektion und muss auf die Wiederherstellung der selbständigen Lebensführung gerichtet sein. Die Maßnahmen müssen indiziert, geeignet und angemessen sein und sollten keinen Schaden zufügen. Sie sollten auf Zustimmung stoßen können, sobald der Patient wieder einwilligungsfähig ist [1].

## „Moral stress“ im Team

Freiheitseinschränkende fixierende/medikamentöse Maßnahmen werden häufig durch Pflegende angewendet [7]. Sich zwischen Maßnahmen zum Schutz vor selbstinduziertem Schaden, also der Eigengefährdung des Patienten und der Würde und dem Respekt vor dem Patienten, entscheiden zu müssen, kann Teammitglieder außerordentlich belasten [29]. Diese moralischen Konflikte („moral stress“), d. h., wenn sich Teammitglieder davon abgehalten fühlen, im Interesse des Patienten zu handeln, sollten benannt und konstruktiv in eine wertschätzende Teamperformance eingebracht werden [30].

## Mögliche Lösungsansätze

### Patientenwille

Ein selbstbestimmtes Leben und Entscheiden unter den Bedingungen einer schweren Erkrankung und intensivmedizinischer Behandlung ist nur sehr begrenzt möglich. Aber auch eingeschränkt oder nicht einwilligungsfähige Patienten sind nicht willenlos. Patienten beschreiben sowohl als verletzend wahrgenommene Situationen, als auch Dankbarkeit, da ihnen geholfen wurde. Diese Ambivalenz macht die große Gefahr von Ausübung von Zwang in einem Abhängigkeitsverhältnis mit dem Risiko, die körperliche und seelische Integrität des Anderen zu verletzen, deutlich [1, 11].

Der Patient ist in seiner Individualität zu sehen und einzubinden

Die Herausforderung für das Team einer hochspezialisierten Intensivstation besteht darin, den Patienten in seiner Individualität zu sehen und einzubinden. Über eine empathische Kommunikation und Teilhabe am Entscheidungsprozess kann eine Einbindung des Patienten in den Entscheidungsprozess, wie sie von vielen Patienten gewünscht wird, erreicht werden [28]. Den Patienten zu informieren, ihm zuzuhören, ihn als Person wahrzunehmen und an Entscheidungen teilhaben zu lassen, stärkt die individuelle Unabhängigkeit des Patienten [20]. Der Patient wird vom Behandlungsobjekt zum Partner [28].

Möglicherweise lassen sich freiheitseinschränkende Maßnahmen oder die Wahrnehmung von Zwang im Rahmen einer intensivmedizinischen Behandlung nicht immer vermeiden. Etablierte Konzepte zur Delirbehandlung, zum Umgang mit Aggressivität und zur Anwendung freiheitseinschränkender Maßnahmen können helfen, Zwang und dessen Wahrnehmung auf der Intensivstation zu minimieren [2, 8].

### Indikation

Neben der Einbindung des Patientenwillens bedarf es besonderer Aufmerksamkeit hinsichtlich der Indikation und der Verhältnismäßigkeit der angewandten freiheitseinschränkenden Maßnahmen. Wenn Alternativen ohne Zwang nicht erreichbar sind, muss reflektiert und dokumentiert werden, mit welcher Maßnahme am wenigsten in die Rechte des Patienten eingegriffen wird [2].

Wichtig erscheint es, jene Freiheitseinschränkungen zu erkennen, die weniger offensichtlich sind, jedoch für den Patienten eine Form von Zwangsmaßnahmen darstellen [18]. Eine individuelle Reflexion über den Patienten als Person, die Indikation und das Therapieziel sowie ein Teamgeist, der sich dieser ethischen Probleme bewusst ist, sind essenzielle Bausteine einer Intensivtherapie, die darauf zielt, Zwang zu vermeiden. Um Zwang und den daraus resultierenden Stress für den Patienten und das Team zu minimieren, sind Schulungskonzepte, spezifische Räume für Reflexionen und eine Teamsupervision zu implementieren.

## Fazit für die Praxis

- Zwang als Überwindung des Willens eines Patienten kann in vielfältiger Form während eines Intensivaufenthalts entstehen.
- Hierzu gehören freiheitseinschränkende Maßnahmen, wie physische Ein‑/Beschränkungen, Einschränkungen durch die Umgebung, medikamentöse und psychologische Einschränkungen.
- Für Zwangsbehandlung existieren juristische Vorgaben und medizinethische Richtlinien.
- Das Erleben von Zwang kann Einfluss auf den Verlauf während und nach einer intensivmedizinischen Behandlung haben.
- Das Spannungsfeld zwischen intensivmedizinischer Behandlung/Fürsorge und eigenen moralischen Werten kann den einzelnen Mitarbeiter und das Team signifikant belasten.
- Den Patienten als Partner einzubinden, erfordert Teamperformance, ethische Reflexion und Wertschätzung, um Zwang auf Intensivstation sowie „moral stress“ zu vermeiden.
- Hier können Ausbildung, Konzepte und Standards zur Reduktion von Zwang auf der Intensivstation hilfreich sein.
